# Intelligent LED Certification System in Mass Production

**DOI:** 10.3390/s21082891

**Published:** 2021-04-20

**Authors:** Galina Malykhina, Dmitry Tarkhov, Viacheslav Shkodyrev, Tatiana Lazovskaya

**Affiliations:** 1 High School of Cyber-Physical Systems and Control, Peter the Great St. Petersburg State Polytechnic University, 195251 Saint Petersburg, Russia; g_f_malychina@mail.ru (G.M.); shkodyrev@mail.ru (V.S.); 2 Scientific and Technological Centre (STC) “Mathematical Modelling and Intelligent Control Systems”, Peter the Great St. Petersburg Polytechnic University, 195251 Saint Petersburg, Russia; dtarkhov@gmail.com; 3 Department of Higher Mathematics, Peter the Great St. Petersburg Polytechnic University, 195251 Saint Petersburg, Russia; 4 Higher School of Software Engineering, Peter the Great St. Petersburg Polytechnic University, 195251 Saint Petersburg, Russia

**Keywords:** efficiency, LED, multi-temporal comparison, cascade neural network, generalized statistical moments

## Abstract

It is impossible to effectively use light-emitting diodes (LEDs) in medicine and telecommunication systems without knowing their main characteristics, the most important of them being efficiency. Reliable measurement of LED efficiency holds particular significance for mass production automation. The method for measuring LED efficiency consists in comparing two cooling curves of the LED crystal obtained after exposure to short current pulses of positive and negative polarities. The measurement results are adversely affected by noise in the electrical measuring circuit. The widely used instrumental noise suppression filters, as well as classical digital infinite impulse response (IIR), finite impulse response (FIR) filters, and adaptive filters fail to yield satisfactory results. Unlike adaptive filters, blind methods do not require a special reference signal, which makes them more promising for removing noise and reconstructing the waveform when measuring the efficiency of LEDs. The article suggests a method for sequential blind signal extraction based on a cascading neural network. Statistical analysis of signal and noise values has revealed that the signal and the noise have different forms of the probability density function (PDF). Therefore, it is preferable to use high-order statistical moments characterizing the shape of the PDF for signal extraction. Generalized statistical moments were used as an objective function for optimization of neural network parameters, namely, generalized skewness and generalized kurtosis. The order of the generalized moments was chosen according to the criterion of the maximum Mahalanobis distance. The proposed method has made it possible to implement a multi-temporal comparison of the crystal cooling curves for measuring LED efficiency.

## 1. Introduction

As digital transformation in the industry associated with the Fourth Industrial Revolution is underway, new information technologies need to be developed, such as computer networks, the internal cloud infrastructure for enterprises, systems of interrelated computing devices, sensors, mechanical and digital machines. One of the directions of these transformations is the implementation of cyber-physical systems connecting physical objects and industrial equipment with computing systems. The revolution in the industry is also associated with the development of a new measuring base, new, more advanced sensors, and technologies. The rapid development of light-emitting diodes (LEDs) in recent years has led to their use in new production systems, workplace lighting systems, alarm systems, high-quality displays, antibacterial optical sensor systems, etc.

The widespread adoption of LED is facilitated by the development of physical foundations for new types of sensors. The publications have described the possibility of a significant increase in the efficiency of light output using UV LEDs based on InGaN/AlGaN [1]. In publication [2], a numerical and experimental study of LEDs with a high reflectivity, which, apparently, is associated with improved current spreading, was carried out. In article [3], a study was made of the influence of GaN/AlGaN layers on the characteristics of ultraviolet LEDs based on GaN, emitting at a wavelength of 375 nm. Publication [4] was devoted to the study of green LEDs InGaN/GaN and their implementation by growing several quantum wells to improve the quality of the crystal.

LED lighting is used in industry for complex manufacturing operations. LED adaptive systems are making a breakthrough in lighting technology in the industry and are quickly becoming one of the most important innovative technologies endorsed by the lighting community [5]. Optical exploration methods in geophysics serve to change traditional geographic approaches [6]. Maintaining the required quality of incident light is critical in plant and microalgae growing processes. Depending on the wavelength of the radiation spectrum and its intensity, the required characteristics of plant growth and their biochemical composition can be provided [7]. LED technology is evolving as a non-thermal food processing technology that uses light energy. LEDs have an antibacterial effect due to photodynamic inactivation of bacteria [8]. The use of LEDs as light sources with the required efficiency is one of the strategies used in the chemical industry [9]. LEDs have a wide range of applications in biomedicine [10]. LED technology is a new technology for non-thermal food processing [11].

These applications lead to rather stringent requirements for the optical characteristics and efficiency of LEDs [12]. High quality is required when creating LED-based calibration lamps [13]. The studies presented in publication [14] proposed a method based on Zernike polynomials for characterizing the photometric values of LEDs and measuring the angular distribution of luminous intensity, total luminous flux, inhomogeneity, anisotropy and direction of the optical axis.

For effective use of LEDs in industrial, medical and telecommunication systems, their effectiveness must be considered. The efficiency of LEDs is an important factor that reflects the ability of these devices to convert electrical energy into optical energy. The publication [15] presented a method for measuring the efficiency of a white LED, based on measuring the power losses arising from the transition to heat. The direct method measures the temperature of a heatsink attached to an LED, and the differential method measures the temperature of two identical heatsinks. The studies were described in [16].

Modern hardware components are used in the system for measuring LED efficiency, which allows reducing the influence of noise to a minimum. However, the measured signals are in the microvolt range and the impact of even the smallest noise is crucial. The comparison results are influenced by intrinsic noise of the measuring channels and external interference. To ensure the reduced error at a level of 1–3%, the value of the signal-to-noise ratio (SNR) in the exponential parts of the signal should be at least 14–20 dB. Reducing the SNR by 3 dB leads to an increase in the reduced error by 1%. Therefore, the most urgent problem is to suppress noise and restore the signal shape, especially in informative areas.

Common noise suppression techniques such as low-pass filtering are known to produce significant waveform distortions. The method of averaging over a sequence of identical pulses leads to heating of the crystal and subsequent distortions of the signal shape. Nonlinear filtering methods are recommended for eliminating noise in the channels of the information-measuring system, because it is necessary to preserve the shape of the signal under study to ensure SNR, as well as to remove noise in the entire frequency range [17].

The peculiarity of adaptive filtering methods is having to search for a reference signal. Adaptive filtering cannot be used if it is impossible to find the reference signal. In view of this, the problem of reconstructing the signal shape can be solved by blind methods for extracting a signal from a mixture of signal and noise. While a whole group of methods and algorithms for blind signal extraction have been developed by now, their direct application does not allow obtaining the highest efficiency of LED [18,19] signal reconstruction. A special algorithm accounting for the statistical properties of signals and noises has to be developed to deal with this issue.

The goal of the study is to develop methods for reconstructing waveforms that help eliminate noise and allow to measure the LED efficiency.

## 2. LED Efficiency Measurement Problem

Currently, there are several approaches to determining the efficiency of LEDs. The analytical calculation approach is the most common. This approach does not consider the entire set of changing parameters [13], which reduces its accuracy. The approach based on the direct measurement of the LED luminous flux [20] also has insufficient accuracy, since it does not consider the conversion of the luminous flux by the LED lens and does not investigate the effect of changing the temperature of the semiconductor crystal. This parameter is the main contributor to the loss of LED efficiency. Evaluating LED performance is important for many industrial and medical applications [21,22].

The efficiency measurement system allows us to explore LEDs regardless of their spectrum. For this, a wide range of settings is provided in the device. Thus, the change in the amplitude of the positive pulse current is provided in the range from 0 to 20 mA. The bias current is adjustable from 0 to 2 mA. Change in the amplitude of the negative pulse current in the range from 0 to 10 mA with a discreteness of 0.1 μA is provided. To ensure the possibility of changing the heating duration of the LED crystal, the device provides the ability to change the pulse duration in the range from 100 μs to 10 s. The system makes it possible to measure not only different types of LEDs, but also to analyze the process of heat transfer from the crystal to the LED bulb and into the environment.

Measurements of efficiency are carried out by the method of multi-time comparison [23,24]. Heat-related losses are the dominant cause of LED power loss. To estimate these losses, we suggest exposing the LED to constant current pulses in the forward positive (I_forv_) direction and in the opposite (reverse, I_rev_) direction. Direct Current (DC) pulses act against a small forward bias current and heat the LED.

When a pulse is applied to the LED in the positive direction, the crystal simultaneously emits light and heats up. Following heating, the LED cools down. When the pulse is applied in the opposite direction, the crystal only heats up. The difference between the power spent on heating the crystal by pulses in the forward and reverse directions allows calculating the power spent on radiation. The normalized power difference characterizes the LED efficiency.

**Figure 1:**
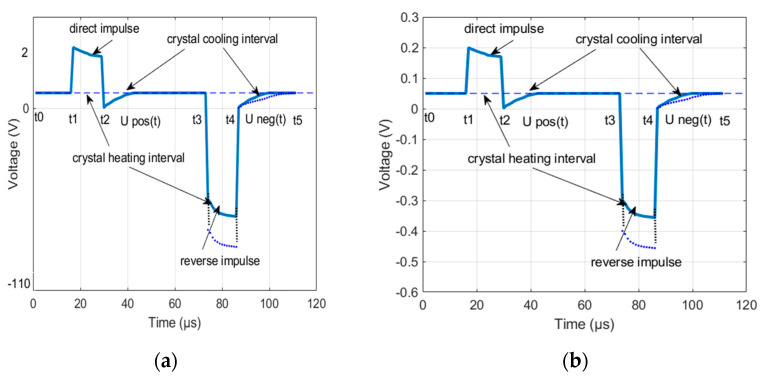
Diagram of voltage variation across an LED, having three heating and cooling periods of the crystal during the LED test. (**a**) The positive signal and the negative signal are presented in the different scales. (**b**) The different gains for positive and negative signals are provided in measuring channels. The gain 1/10 was established for the positive signal and 1/100 for the negative signal.

The method consists in the cooling curves of the LED with a positive and negative pulse. Constant current pulses are applied to the LED under investigation. The LED voltage amplifier forms a diagram of the voltage variation across the LEDs. For further signal processing, various gains of the amplifiers of the measuring channel are provided. The gain was 1/10 for the positive signal and 1/100 for the negative signal. The converted LED voltage measurement signal is shown in Figure 1. The time on the abscissa axis is shown in relative units, since the diagram shows the general principle, it does not delve into the details of a particular experiment. The cooling curves for the positive and negative impulses did not match in the first and second periods. It was possible to achieve equal cooling curves in the third test period. The first curve was formed upon exposure to a current pulse of positive polarity, at the moment *t*_1._ In this case, heating of the semiconductor crystal and radiation were observed. At time *t*_2_, the action of the current pulse stopped and the LED crystal cooled down, as shown by the curve $U_{pos}\left(t\right)$. A current pulse of negative polarity acted at time *t*_3_, and only heating of the semiconductor crystal was observed. The action of the pulse ceased at time *t*_4_, and the LED crystal cooled down, as shown by the curve $U_{neg}\left(t\right)$.

To determine the efficiency, the reverse current $I_{inv}$ should be adjusted so that the norm of the difference between the curves $U_{neg}\left(t\right)$ and $U_{pos}\left(t\right)\;$ does not exceed the given mismatch error $\varepsilon \leq \int_{0}^{T}{\left(U_{pos}\left(t\right)-U_{neg}\left(t\right)\right)}^{2}dt$, where $T=t_{3}-t_{2}=t_{5}-t_{4}$.

Provided that compensation has been achieved, the LED efficiency value *η* can be calculated using the following formula:

(1) $$\eta =\frac{\int_{t1}^{t2}I_{pos}\left(t\right)U_{pos}\left(t\right)dt-\int_{t3}^{t4}I_{neg}\left(t\right)U_{neg}\left(t\right)dt}{\int_{t1}^{t2}I_{pos}\left(t\right)U_{pos}\left(t\right)dt}$$

where $I_{pos}\left(t\right),U_{pos}\left(t\right),\;I_{neg}\left(t\right),\;U_{neg}(t)$ are the electric current and voltage at which the LED is exposed to positive or negative pulses.

The mismatch of cooling curves $U_{pos}(t)\;$and$\;U_{neg}(t)$ corrupted by noise is represented by formula

(2) $$\begin{array}{ll} e & =\int_{0}^{T}{\left(\left(u_{pos}\left(t\right)+n_{1}\left(t\right)\right)-\left(u_{neg}\left(t\right)+n_{2}\left(t\right)\right)\right)}^{2}dt\\ & =\int_{0}^{T}{\left(\left(u_{pos}\left(t+t_{1}\right)+n_{1}\left(t+t_{1}\right)\right)-\left(u_{neg}\left(t+t_{3}\right)+n_{2}\left(t+t_{3}\right)\right)\right)}^{2}dt \end{array}$$

where $n_{1}\left(t\right),n_{2}\left(t\right)$ correspond to the noise acting on the cooling curves.

Ensemble averaging over a sequence of positive and negative pulses allows obtaining the average value of mismatch:

(3) $$\begin{array}{ll} \varepsilon & =E\left\{\int_{0}^{T}{\left(\left(u_{pos}\left(t+t_{1}\right)+n_{1}\left(t+t_{1}\right)\right)-\left(u_{neg}\left(t+t_{3}\right)+n_{2}\left(t+t_{3}\right)\right)\right)}^{2}dt\right\}\\ & =E\left\{\int_{0}^{T}{\left(\left(u_{pos}\left(t+t_{1}\right)-u_{neg}\left(t+t_{1}\right)\right)+\left(n_{1}\left(t+t_{1}\right)-n_{2}\left(t+t_{3}\right)\right)\right)}^{2}dt\right\}. \end{array}$$

It consists of three terms:

(4) $$\begin{array}{ll} \varepsilon = & \int_{0}^{T}{\left(u_{pos}\left(t+t_{1}\right)-u_{neg}\left(t+t_{1}\right)\right)}^{2}dt+E\left\{\int_{0}^{T}{\left(n_{1}\left(t+t_{3}\right)-n_{2}\left(t+t_{3}\right)\right)}^{2}dt\right\}\\ & -2\cdot E\left\{\int_{0}^{T}\left(u_{pos}\left(t+t_{1}\right)-u_{neg}\left(t+t_{1}\right)\right)\cdot \left(n_{1}\left(t+t_{1}\right)-n_{2}\left(t+t_{3}\right)\right)dt\right\}. \end{array}$$

Supposing that the signal and the noise are uncorrelated,

(5) $$E\left\{\int_{0}^{T}\left(u_{pos}\left(t+t_{1}\right)-u_{neg}\left(t+t_{1}\right)\right)\cdot \left(n_{1}\left(t+t_{1}\right)-n_{2}\left(t+t_{3}\right)\right)dt\right\}=0,$$

We obtain the following equation of mismatch average value:

(6) $$\begin{array}{ll} \varepsilon = & \int_{0}^{T}{\left(u_{pos}\left(t+t_{1}\right)-u_{neg}\left(t+t_{1}\right)\right)}^{2}dt+E\left\{\int_{0}^{T}{\left(n_{1}\left(t+t_{1}\right)\right)}^{2}dt\right\}\\ & +E\left\{\int_{0}^{T}{\left(n_{2}\left(t+t_{3}\right)\right)}^{2}dt\right\}+2\\ & \cdot E\left\{\int_{0}^{T}\left(n_{1}\left(t+t_{1}\right)\cdot n_{2}\left(t+t_{3}\right)\right)dt\right\} \end{array}$$

where $r\left(t_{3}-t_{1}\right)=E\left\{\int_{0}^{T}\left(n_{1}\left(t+t_{1}\right)\cdot n_{2}\left(t+t_{3}\right)\right)dt\right\}$ is the autocorrelation of noise. The following equation is true for the previous two terms:

(7) $$E\left\{\int_{0}^{T}{\left(n_{1}\left(t+t_{1}\right)\right)}^{2}dt\right\}=E\left\{\int_{0}^{T}{\left(n_{2}\left(t+t_{3}\right)\right)}^{2}dt\right\}=T\sigma^{2}{}_{n}\;\;$$

where $\sigma_{n}$ is the standard deviation of noise.

The average value of mismatch for an analog signal can be written as an integral:

(8) $$\varepsilon =\int_{0}^{T}{\left(u_{pos}\left(t+t_{1}\right)-u_{neg}\left(t+t_{3}\right)\right)}^{2}dt+2T\sigma_{n}^{2}.$$

The average value of mismatch for a discrete signal is given in decimal notation:

(9) $$\varepsilon =\overset{N-1}{\underset{\tau =0}{\sum }}{\left(u_{pos}\left(\tau +\tau_{1}\right)-u_{neg}\left(\tau +\tau_{3}\right)\right)}^{2}+2N\sigma_{\mathrm{III}}^{2}+2Nr\left(\Delta \tau \right)$$

where ΔT is the sampling period, t=τΔT, N=TΔT, $\Delta \tau =\tau_{3}-\tau_{1}$.

The average value of mismatch depends on *N* and increases proportionally to the duration of the cooling interval. The normalized average value of mismatch may be estimated by the formula:

(10) $$\frac{\varepsilon }{N\sum_{\tau =0}^{N-1}u_{pos}{\left(k+k_{1}\right)}^{2}}=\frac{1}{N}\frac{\sum_{\tau =0}^{N-1}{\left(u_{pos}\left(k+k_{1}\right)-u_{neg}\left(k+k_{3}\right)\right)}^{2}}{\sum_{\tau =0}^{N-1}u_{pos}{\left(k+k_{1}\right)}^{2}}\;+2\frac{\sigma_{n}^{2}}{\sum_{\tau =0}^{N-1}u_{pos}{\left(k+k_{1}\right)}^{2}}+2\frac{r_{n}}{\sum_{\tau =0}^{N-1}u_{pos}{\left(k+k_{1}\right)}^{2}}.$$

The first term after reaching a match is zero. Assuming an acceptable mismatch error of $10^{-2}$, the corresponding SNR can be calculated by using the equations:

(11) 10⋅lg(u22σn2+u22rn)+10ln12=10⋅lg110−2

where $u^{2}=\sum_{\tau =0}^{N-1}u_{pos}{\left(k+k_{1}\right)}^{2}$,

(12) $$SNR=10\cdot \mathrm{lg}\frac{u^{2}}{\sigma_{\mathrm{III}}^{2}}=10-10\mathrm{lg}\frac{1}{2}-10\mathrm{lg}\frac{\rho }{\rho +1}$$

where ρ=rnσn2. The allowable values are SNR=23 db, given that ρ=0, and SNR=28 db, given that ρ=0.5.

Noises in the measuring channels do not allow to determine a moment when compensation appears. Let us introduce the admissible mismatch error, which, for example, is equal to $\delta =10^{-2}$; then the corresponding admissible SNR value is determined using the equation:

(13) 10⋅lg(u22σn2+u22rn)+10ln12=10⋅lg1δ=10⋅lg110−2

where $u^{2}=\sum_{\tau =0}^{N-1}u_{pos}{\left(t+t_{1}\right)}^{2}$, $\sigma_{n}$ is the standard deviation of noise,$\;r_{n}=r_{n}\left(t_{3}-t_{1}\right)$ is the autocorrelation function of noise

(14) $$SNR=10\cdot \mathrm{lg}\frac{u^{2}}{\sigma_{\mathrm{III}}^{2}}=10-10\mathrm{lg}\frac{1}{2}-10\mathrm{lg}\frac{\rho +1}{\rho }$$

where ρ=rnσn2.

Evaluation of efficiency is most sensitive to the accuracy of determining the cooling curve U_neg_ (t) and to noise in the flat sections of pulses. Noise in these areas must be carefully suppressed for automatic testing of any type of LED. It is possible to estimate the effect of noise on the error in determining the coincidence of the cooling curves of crystals.

If we assume that the average relative error in the mismatch of cooling curves is 1%, and *ρ* = 0–0.5, then the SNR should be at a level of 19–23 dB. If the requirement for the average relative error of mismatch is tightened to 0.1%, then an SNR of 29–33 dB should be provided. Thus, we determined a condition under which it makes sense to measure the LED efficiency, namely, that it is necessary to provide an SNR of at least 30 dB.

The modern electronic components that are used to design the system for measuring LED efficiency allow to reduce noise to a minimum. However, the value of the SNR is still unacceptable for measuring the LED efficiency. The rest of noise in the signal frequency domain results in distortion of signal.

Noise-suppressing methods such as low-pass filtering lead to distortion of the signal waveform. The method of averaging identical pulses (Figure 1) from a sequence of pulses results in overheating the crystal and, therefore, distorting the signal waveform. The method of linear adaptive filtering cannot be applied because of the lack of a reference signal. Thus, the method of sequential blind extraction of signal from a mixture of signal and noise is best-suited for the problem of noise suppression during LED testing. This method is implemented by organizing at least two channels containing a mixture of signal and noise. In practice, there is often uncorrelated Gaussian noise in the two channels organized. This problem can also be solved by adding a measuring channel, which complicates the electronic measurement circuit.

There are various approaches to extracting signal from a mixture of signal and noise. This includes independent component analysis, blind signal separation and blind signal extraction methods [25,26,27,28]. The blind signal extraction method has an advantage over other known methods for reducing noise in the LED certification system. This method is implemented by a cascade neural network. Because the curve of heating and cooling is extracted and there is no need to extract noise, a single-layer neural network may be used. Thus, the goal is to develop such a combined scenario for processing the LED heating and cooling signals that provide the SNR of at least 30 dB on the cooling curve.

## 3. Reconstruction of Signal from a Mixture of Signal and Noise

### 3.1. Blind Signal Extraction Problem

Mixing models of signal and noise components $s\left(t\right)={\left[s_{1}\left(t\right),s_{2}\left(t\right),\ldots ,s_{m}\left(t\right)\right]}^{T}$ using a nonsingular matrix **H** [29,30] affects the signal **x**(*t*) = **H s**(*t*). The resulting mixture model is fed to the system input. If the signal components are correlated, it is advisable to apply a decorrelated transform. As a result of mixing, the signal $x\left(t\right)={\left[x_{1}\left(t\right),x_{2}\left(t\right),\ldots ,x_{m}\left(t\right)\right]}^{T}$. The separating matrix **W** should be constructed to obtain a vector signal ***y***(*t*), which is an estimate of the unknown signal vector ***s***(*t*) for a mixture of signals y(t)=Wx(t) observed. Therefore, reconstruction of the correct waveform in the cooling sections during LED testing can be formulated as a problem of estimating the vector ***y***(*t*) of the original signal by searching for the operator **W** to back-mix the signal and the noise.

Since only one component of the mixture is of interest, i.e., the useful signal in cooling sections, we suggest to apply the method of the sequential extraction of useful signal. This method can be implemented by a neural network whose weight matrix ***W*** is selected during the adaptation process. In this case, ***W*** consists of *m* vectors ***W*** = [***w***_1_, ***w***_2_, ..., ***w***_m_], each vector is adapted in one cascade of a neural network.

### 3.2. Estimation of Statistical Characteristics of Signal and Noise

It is possible to extract the signal from a mixture of signal and noise if the signal and the noise have well-differentiated statistical characteristics that are high-order statistical moments. For proof, we examined the statistical characteristics of signal and noise. The study indicated that the probability density function PDF of the signal is characterized by high skewness and high kurtosis. On the other hand, the PDF of noise is close to Gaussian, while the noise skewness is close to zero. A more detailed study of the PDF of noise was performed using a generalized Gaussian PDF. Generalized Gaussian PDF is a family of PDFs of various shapes that are characterized by three parameters: the mathematical expectation ($m_{x}$), the standard deviation ($\sigma_{x}$), and the shape parameter (α) [31].

The following formula defines a generalized Gaussian PDF:

(15) $$f(x)=\frac{\alpha }{2\lambda \sigma \Gamma {\left(\frac{1}{\alpha }\right)}_{x}}\exp\left(-{\left|\frac{x-m_{x}}{\lambda \sigma_{x}}\right|}^{\alpha }\right),$$

(16) $$\Gamma \left(\alpha \right)=\overset{\infty }{\underset{0}{\int }}x^{\alpha -1}\exp\left(-x\right)dx$$

where Γ(α) is the gamma function.

The generalized Gaussian distribution has such properties as the simplicity of mathematical description, the possibility of wide variation of shape from a peaked to rectangular uniform distribution, the convenience of statistical estimation of parameters, making this family a favorable choice for describing the PDF of signal and noise.

The results of evaluating the four statistical moments of noise confirmed that the PDF of noise is close to Gaussian, since the parameter α=2.08 was close to the parameter α of the Gaussian distribution (α=2). Signal and noise PDFs differed significantly, suggesting that reconstruction of the waveform using the statistical moments of the PDF skewness or kurtosis as an objective function was effective.

The statistical moments of signal and noise were estimated using an electronic measuring circuit and the process of comparing the crystal cooling curves. The calculated values of the mathematical expectation, variance, skewness and kurtosis of the signal are presented in Table 1.

Statistical moments indicated that the empirical PDF of the signal could not be represented by a Gaussian distribution, since the PDF was asymmetric and peaked.

The statistical moments of noise were estimated using the electronic circuit. Four realizations of noise are given in Table 2. The generalized Gaussian distribution allowed for variation of shape in a wide range from peaked to squared.

The statistical moments given in Table 3 show that the PDF of noise approximated to the generalized Gaussian form for four PDF realization ranges within *α* = 1.25 (Laplacian) and *α* = 2.08 (Gaussian).

As shown in Table 4 and Table 5, the noise was characterized by a symmetrical distribution close to an exponential with the kurtosis of 2.42–6.77. The signal had a significant skewness of 4.04 and kurtosis of 17.3. This fact made it possible to use a method based on normalized statistical moments to extract a useful test signal from a mixture of signal and noise.

Comparing the statistical moments of signal and noise given in Table 1 and Table 2, we can see that the difference between the statistical moments of the first and second order was less than the difference between excesses, the statistical moments of a higher order. Therefore, higher-order moments were better suited to solving the problem. However, it was also necessary to compare the errors of estimating the statistical moments, which were larger for higher-order moments [32,33,34,35,36].

### 3.3. Combining Skewness and Kurtosis

Let us consider the case when the objective function for signal extraction uses skewness, the normalized third central statistical moment given by the formula:

(17) $$J\left(w\right)=-\frac{1}{3}\left|k_{3}\left(y\right)\right|=-\frac{\beta }{3}k_{3}\left(y\right)$$

where *y* is the signal of interest, $k_{3}\left(y\right)=\frac{E\left\{y^{3}\right\}}{E^{2}\left\{y^{3/2}\right\}}$ is the normalized statistical moment of skewness, *β* is the parameter accounting for the sign of skewness. A computational experiment showed that using the objective function (17) allows to reconstruct the signal shape, provided that SNR = 16 dB is obtained.

Further, we considered using an objective function based on the fourth central statistical moment, which is determined by the formula:

(18) $$J\left(w\right)=-\frac{1}{4}\left|k_{4}\left(y\right)\right|=-\frac{\beta }{4}k_{4}\left(y\right)$$

where $k_{4}\left(y\right)=\frac{E\left\{y^{4}\right\}}{E^{2}\left\{y^{2}\right\}}$ is the normalized kurtosis.

If we combine two statistical moments of the kurtosis and the skewness into one objective function with weight coefficients $\lambda_{1}$ and $\lambda_{2}$, $J\left(w\right)=-\lambda_{1}\frac{\beta_{1}}{3}k_{3}\left(y\right)-\lambda_{2}\frac{\beta_{2}}{4}k_{4}\left(y\right)$, then choosing the appropriate $\lambda_{1}$ and $\lambda_{2}$, $\lambda_{1}\geq 0,\;\lambda_{1}\geq 0,$ $\lambda_{1}+\lambda_{2}=1,$ the better results are obtained. The optimization of the objective function with $\lambda_{1}=\lambda_{2}=0.5$ was carried out using the gradient descent method according to the following formula:

(19) dwdt=μβ1λ1(m232m23E{y2x}−m3m212m23 E{yx})+μβ2λ2(1m22E{y3x}−m4m23 E{yx})

where *m_p_*, *p* = 1, 2, 3, 4, are the statistical moments of order *p*.

The proposed approach to signal extraction from LED testing has a simple explanation. It is known from the theory of probability that the sum of independent random variables with approximately the same scales has a distribution close to Gaussian. Therefore, the sum of several random signals and noises has a distribution that is closer to Gaussian than any of the original random signals. Consequently, the problem of extracting a signal from the mixture is reduced to find the vector $w_{i}$ which maximizes the difference between the PDF of the output signal and the Gaussian. The absolute value of the normalized kurtosis and the normalized skewness is the simplest measure of the non-Gaussianity of the extracted signal. This follows from Equation (19), which minimizes the objective function based on the normalized kurtosis and skewness.

**Figure 2:**
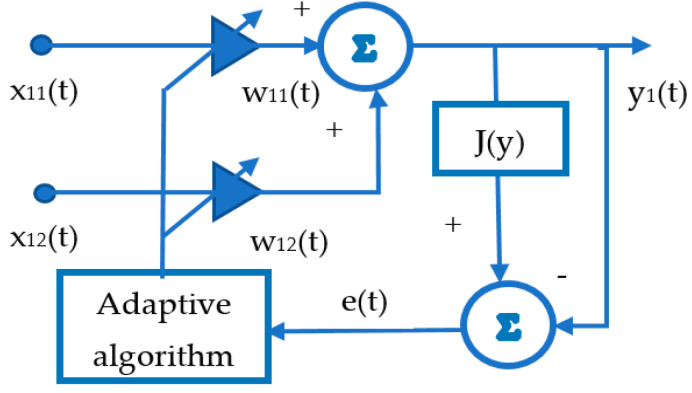
Single-layer neural network training.

Solving the optimization problem can be considered as the process of adapting the coefficients $w_{11},\;w_{12}$ of a single-layer neural network using the gradient descent method. An attractive feature is training the neural network without a supervisor. A diagram of neural network training is shown in Figure 2 .

The activation function in the neuron considered is implicit. The neural network activation function for the combined method is:

(20) $$\varphi \left(y\right)=\left[\beta_{1}\left(\frac{m_{2}^{\frac{3}{2}}}{m_{2}^{3}}E\left\{y^{2}\right\}-\frac{m_{3}\cdot m_{2}^{\frac{1}{2}}}{m_{2}^{3}}E\left\{y\right\}\right)+\beta_{2}\left(\frac{1}{m_{2}^{2}}E\left\{y^{3}\right\}-\frac{m_{4}}{m_{2}^{3}}E\left\{y\right\}\right)\right].$$

**Figure 3:**
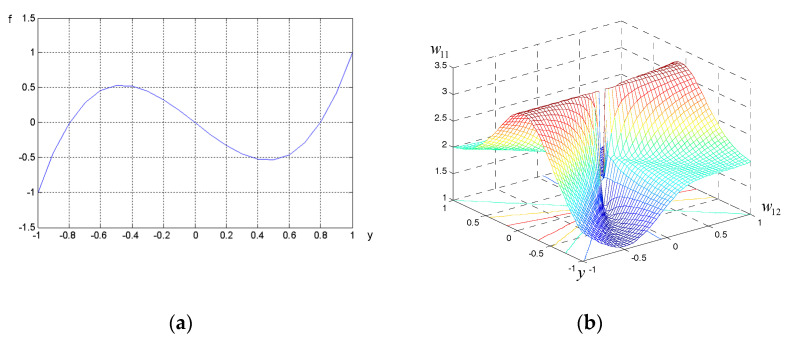
Activation function (**a**) and objective function surface (**b**).

For this reason, the activation function does not appear in Figure 3. The activation function changes during the process of adaptation of neuron weights. The activation function of the combined signal extraction method and the objective function surface are shown in Figure 3.

Adaptation of the neural network for sequential signal extraction is

(21) w(k+1)=w(k)+μ(k) φ(y(k)) x(k)

where *μ*(*k*) is the learning rate. This value provides a trade-off between the signal extraction accuracy and the adaptation time. In the simulation experiment, we took *μ*(*k*) = 0.01.

We implemented online neural network training using the gradient descent algorithm (21) and the adaptive activation function (20).

### 3.4. Determining the Order of Statistical Moments

The statistical moments of higher orders are more sensitive to the shape of the signal and noise distribution; therefore, it seems promising to apply them. The error in estimating statistical moments based on signal and noise samples is important.

To calculate the variance *D* of the estimate of the central statistical moment of order *p*, for *p* = 1–6, according to the formula $D_{m\left(p\right)}=\frac{1}{n}\mu_{2p}-\mu_{p}^{2}+p^{2}\mu_{2}\mu_{p-1}^{\prime }-2p\mu_{p-1}\mu_{p+1}$, it is necessary to have statistical moments up to the 12th order inclusive. The resulting standard deviations determined for the estimates of statistical moments for signal and noise are presented in Table 3.

**Figure 4:**
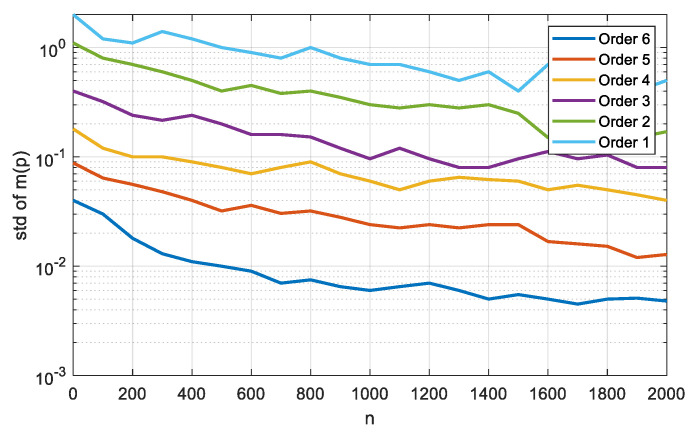
Dependence of root-mean-square error of kurtosis on the volume of sample.

As shown in Figure 4, high-order statistical moments have large estimation errors. Large data samples have to be accumulated to estimate them. Thus, it is necessary to determine the order of statistical characteristics that are better suited for constructing the objective function [37,38,39]. We used the generalized kurtosis and the generalized skewness based on the statistics of arbitrary order for the objective function. It is advisable to choose their order based on analysis of two indicators: the sensitivity to the PDF shape and the error of statistical estimation.

Generalized skewness and generalized kurtosis of order *p* and *q* are determined by the formulas:

(22) $$A_{pq}\left\{y\right\}=\frac{E\left\{\mathrm{sign}\left(y\right){\left|y\right|}^{p}\right\}}{E^{q}\left\{y^{p/q}\right\}},$$

(23) $$k_{pq}\left\{y\right\}=\frac{E\left\{{\left|y\right|}^{p}\right\}}{E^{q}\left\{{\left|y\right|}^{p/q}\right\}}.$$

The objective function of the combined method based on generalized moments is written as

(24) $$J_{p1q1p2q2}(w)=\lambda_{1}\frac{1}{p_{1}}A_{p1q1}\left(w^{T}x\right)+\lambda_{2}\frac{1}{p_{2}}k_{p2q2}\left(w^{T}x\right)$$

where *p*_1_, *q*_1_ are the indicators of the order of generalized skewness, *p*_2_, *q*_2_ are the indicators of the order of generalized kurtosis.

Taking these indicators into account, the formula for adapting the neural network using the gradient descent method is as follows:

(25) $$\begin{array}{ll} \frac{dw}{dt}= & \lambda_{1}\mu E\left\{\mathrm{sign}\left(y\right)\cdot {\left|y\right|}^{p_{1}-1}x_{1}\right\}\frac{1}{m_{\frac{p1}{q1}}^{q1}\left(y\right)}+\lambda_{2}E\left\{{\left|y\right|}^{p2-1}x\right\}\cdot \frac{1}{m_{\frac{p2}{q2}}^{q2}\left(y\right)}-\frac{m_{p1}\left(y\right)}{m_{\frac{p1}{q1}}^{q1+1}\left(y\right)}\\ & \cdot E\left\{\mathrm{sign}\left(y\right){\left|y\right|}^{p1/q1-1}x\right\}-\frac{m_{p2}\left(y\right)}{m_{p2/q2}^{q2+1}\left\{{\left|y\right|}^{p2/q2}\right\}}\cdot E\left\{\mathrm{sign}\left(y\right){\left|y\right|}^{p2/q2-1}x\right\}. \end{array}$$

Further study was aimed at selecting the appropriate order of statistical moments. The error of generalized statistical moments estimation depends on the statistical sample size and the orders $p_{1},$ $p_{2},$ $q_{1},$ $q_{2}$ of the statistical moments. Normalized statistical moments $A_{pq}$ and $k_{pq}$ are stochastic variables with the PDF. To increase the speed and the quality of adaptation of the neural network, the distance between the generalized skewness of the signal and the noise as well as the distance between the generalized kurtosis of signal and noise should be large. To estimate the distance, we used the Mahalanobis measure between the estimates of the statistical moments of signal and noise of orders *p* and *q*:

(26) $$D=\frac{1}{2}\frac{\left(\sigma_{S}^{2}-\sigma_{N}^{2}\right)\left(\sigma_{N}^{2}-\sigma_{S}^{2}\right)}{\sigma_{N}^{2}\sigma_{S}^{2}}+\frac{\sigma_{N}^{2}+\sigma_{S}^{2}}{\sigma_{S}^{2}\sigma_{N}^{2}}{\left(m_{S}-m_{N}\right)}^{2}$$

where $\sigma_{S}$, $\sigma_{N}$ are the standard deviations of the statistical moments $A_{pq}$ and $k_{pq}$ of signal and noise, respectively; $m_{S}$, $m_{N}$ are the expectation of the statistical moments $A_{pq}$ and $k_{pq}$ of signal and noise, respectively.

**Figure 5:**
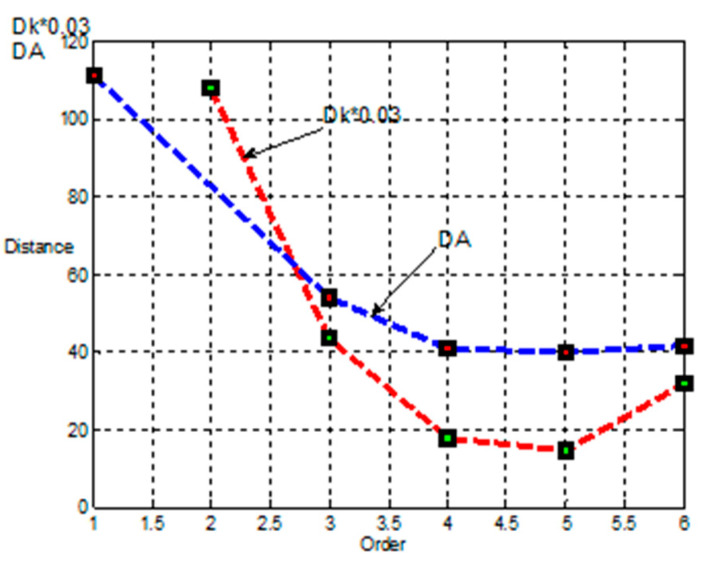
Dependencies of Mahalanobis distance on the order of statistical moments.

Dependences of the Mahalanobis distance for skewness $D_{A}$ and kurtosis $D_{k}$ on the order of statistical moments calculated by Equation (26) are shown in Figure 5.

The obtained dependences of the Mahalanobis distance made it possible to choose the order of kurtosis and skewness for implementation in the combined method of signal extraction. Indeed, the choice of generalized kurtosis and generalized low-order skewness is efficient, since the moments have not only a smaller estimation error but also a larger Mahalanobis distance.

Based on the analysis performed, we suggest to construct an objective function using generalized skewness with the parameters $p_{1}=2,\;q_{1}=1$ and generalized kurtosis with the parameters p2=1, q2=12.

The algorithm for reconstructing the signal waveform for LED certification includes the following steps:

1. Obtain the measured voltage values during heating and cooling of the LED at two outputs of the electronic circuit;
2. Calculate the skewness of order *p*_1_ = 2, *q*_1_ = 1 and the kurtosis of order *p*_2_ = 1, *q*_2_ = 1⁄2;
3. Calculate the activation function by Equation (20);
4. Implement the adaptation of the neural network cascade (25);
5. Fit the activation function and repeat Step 4;
6. Check the end condition for the adaptation $\varepsilon \leq \int_{0}^{T}{\left(U_{+}\left(t\right)-U_{-}\left(t\right)\right)}^{2}dt$;
7. If the condition for ε is satisfied, then calculate the LED efficiency by Equation (2);
8. If the condition is false, then change the current magnitude and go to Step 1.

## 4. Modeling the Signal Waveform Reconstruction Process

**Figure 6:**
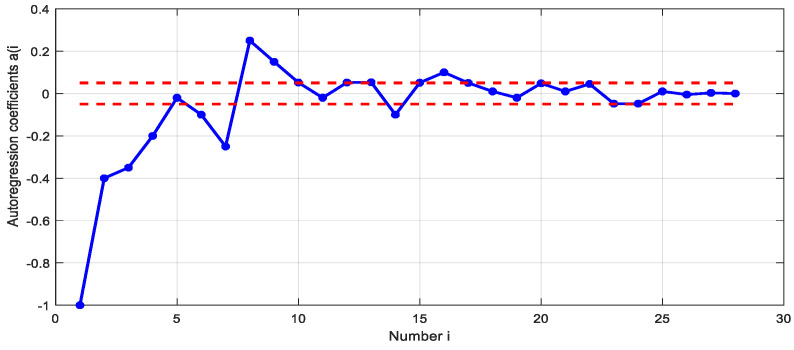
Estimation of autoregressive coefficients (blue line) with confidence intervals (red dashes).

A simulation model was used to study the proposed method. The signal is a deterministic function that changes when cooling curves are fitted. Noise is represented by a model of decreasing power spectral density. Identifying the noise *n*(*t*), we found that it can be described by the autoregressive process of order *p* [40,41]:

(27) $$n\left(t\right)=a_{0}+\overset{P}{\underset{i=0}{\sum }}a_{i}n\left(t-i\right)+e\left(t\right)$$

where $a_{i}$ are the parameters of the autoregressive model, *p* = *18* is the order of the autoregressive model, e(t) is white noise. The autoregression coefficients and the ranges of its estimation error are shown in Figure 6.

A signal extraction procedure was simulated to compare the variants of the suggested target functions based on skewness, kurtosis, and a combination of skewness and kurtosis. The signal model was represented by a sequence of pulses shown in Figure 1, and the noise model was represented by the autoregressive process (27).

**Figure 7:**
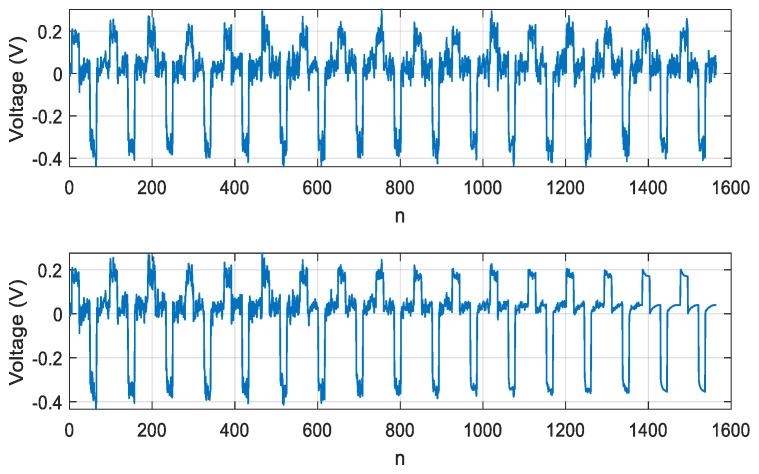
Dependence of extracted signal on discrete time n=tΔt, Δt=1μs, implying an extraction based on skewness.

Using the objective function (18) at $\lambda_{1}\geq \;\lambda_{1}=1,\;\lambda_{2}=0,$ we obtained the result of extracting a signal with the SNR of 16 dB, at 15 test periods of the LED. The result of signal extraction is shown in Figure 7.

**Figure 8:**
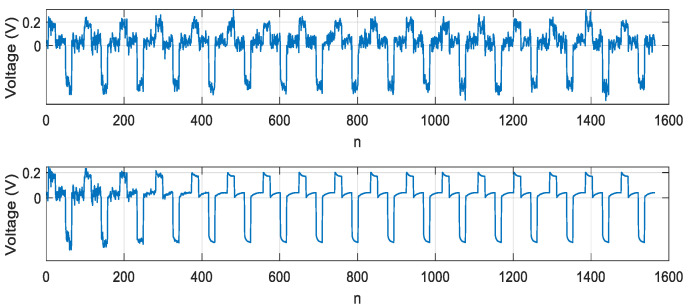
Dependence of extracted signal on discrete time n=tΔt, Δt=1μs, implying an extraction based on kurtosis.

When using the objective function (18) with $\lambda_{1}=0,\;\lambda_{2}=1,$ based on normalized kurtosis, we obtained the result of extracting a signal with the SNR of 23 dB, with 5 LED test periods. The result of signal extraction is shown in Figure 8.

**Figure 9:**
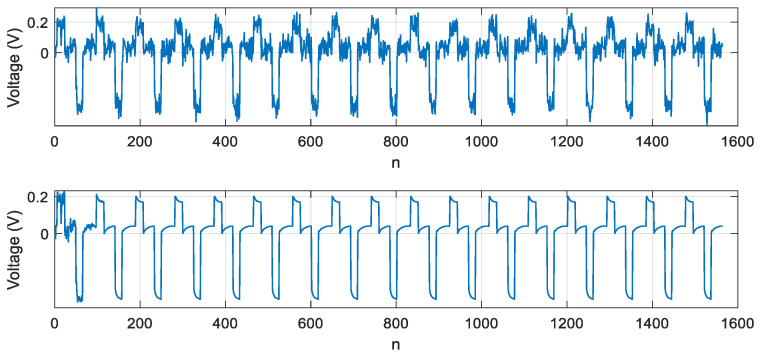
Dependence of the extracted signal on discrete time n=tΔt,Δt=1μs, implying an extraction obtained using the combined criterion based on skewness and kurtosis.

The combined method was based on normalized skewness and kurtosis with the weights $\lambda_{1}=0.5,\;\lambda_{2}=0.5$, the signal can be reconstructed with SNR = 28 dB. Neural network adaptation was performed over two periods. The result of signal extraction is shown in Figure 9.

The algorithm was further improved using the objective function (25) based on the statistical moments of the order that was optimal for solving our problem. These moments are $p_{1}=2,\;q_{1}=1$ for generalized skewness and $p_{2}=1,\;q_{2}=1/2$ for generalized kurtosis.

Applying a step-by-step learning algorithm of second order, for example, the Levenberg–Marquardt (LM) or Broyden–Fletcher–Goldfarb–Shanno (BFGS) algorithm, yielded better results.

**Figure 10:**
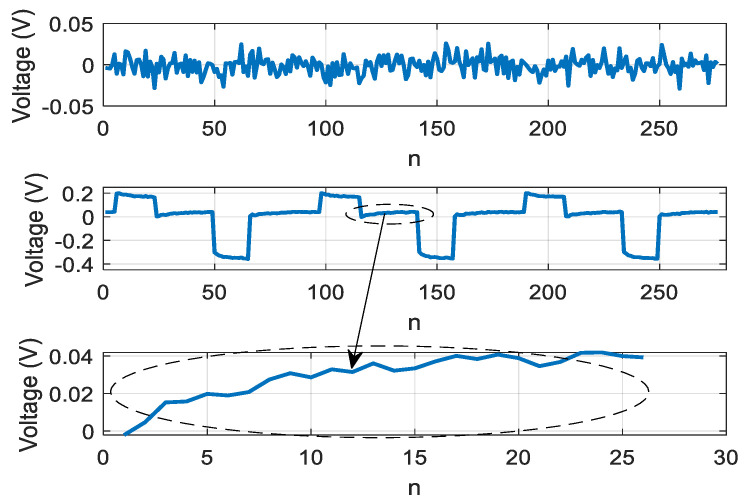
Dependence of the extracted signal on discrete time n=tΔt, Δt=1μs, implying an extraction obtained using the combined criterion based on skewness and kurtosis.

Figure 10 shows the results of signal extraction obtained by executing an algorithm based on relation (18) and step-by-step training using the BFGS method. SNR = 16 dB was consequently achieved in the local sections of the cooling curves of the LED crystal.

**Figure 11:**
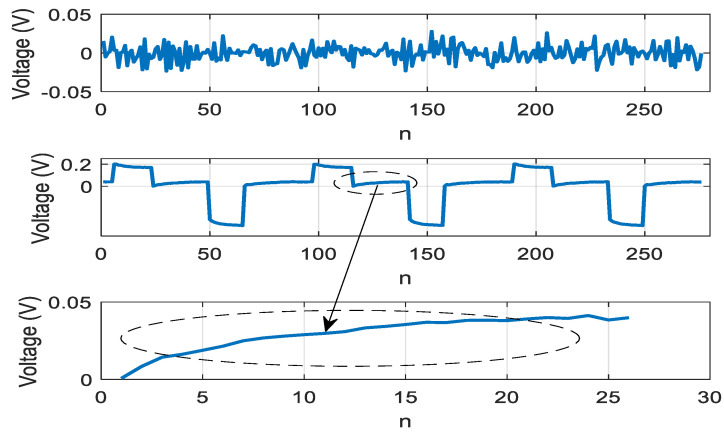
Dependence of the extracted signal on discrete time n=tΔt, Δt=1μs, implying an extraction obtained using the combined criterion based on generalized skewness, generalized kurtosis and second order adaptation algorithm.

Figure 11 shows the result of signal extraction by executing an algorithm based on Equation (25) and the training algorithm based on the BFGS method. SNR = 41 dB was consequently achieved in the local sections of the cooling curves of the LED crystal.

## 5. Discussion

To suppress noise in the process of determining the LED efficiency, attempts were made to apply filters in the power supply circuit, galvanic isolation of analog and digital equipment, electromagnetic shielding of the signal cable and analog equipment, and smoothing low-frequency filters. An SNR of −14 dB was obtained through using noise suppression tools.

**Figure 12:**
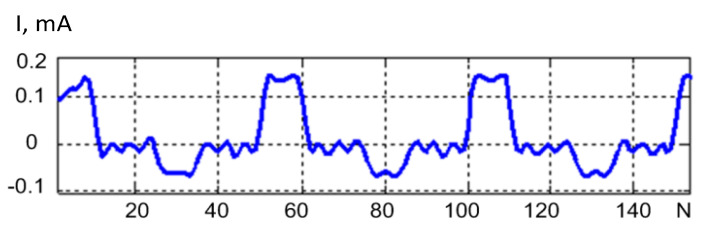
Noise suppression using a 9th order Butterworth filter with a cutoff frequency of 330 kHz.

Digital low-pass filters with finite impulse response (FIR), and Butterworth, Chebyshev, Bessel [42,43] filters with infinite impulse response (IIR), were considered at the next stage. The low-pass FIR filters produced some waveform distortion but did not suppress noise in the LED cooling curves. Applying the low-pass FIR filter yielded an SNR = −6 dB, while an SNR of −7 dB was obtained in the case of the IIR filter. The result of applying the Butterworth filter is shown in Figure 12.

**Figure 13:**
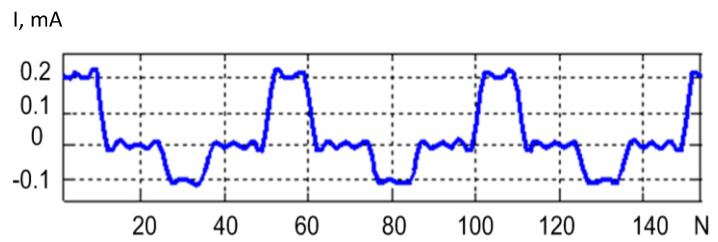
Noise suppression using a 9th order Type I Chebyshev filter with a cutoff frequency of 330 kHz.

The Type I Chebyshev filter of the 9th order with the cutoff frequency of 330 kHz allowed achieving SNR = −6 dB (see Figure 13).

**Figure 14:**
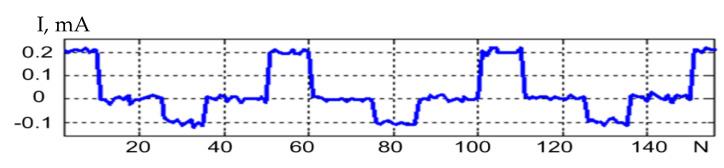
Noise suppression via averaging of corresponding pulses.

Noise suppression by averaging the sequence of identical signal pulses [43] also did not give good results. Calculations indicated that the number of identical pulses required for the averaging operation must be at least 100. When such a number of pulses accumulates, the crystal is heated, which did not allow to implement this method. The result of noise suppression by averaging over five periods yielded an SNR = −4 dB (see Figure 14).

Thus, classical noise suppression methods did not provide the required SNR. Using adaptive filters was complicated because a reference channel had to be organized Modeling confirmed that the proposed adaptive method for signal extraction provided a result that allowed testing the LED efficiency.

This method was complex to implement since uncorrelated noise appeared in two measurement channels. The challenge presented by having to compensate for the channel’s own noise could be overcome by creating a third measuring channel. We simulated the signal extraction procedure in the case when the proportion of uncorrelated white noise was 7%, making it possible to obtain satisfactory results.

## 6. Conclusions

The suggested method used a single-layer neural network to extract the signal in the cooling sections of the LED upon exposure to pulses of positive and negative polarities. This method performed simultaneous comparison of the cooling curves of the crystals and calculation of the LED efficiency at the onset of compensation. Using an objective function for training a neural network that combines the normalized statistical moments of signal and noise PDFs gives a pronounced effect during signal extraction, particularly if the order of statistical moments is specially adapted to solving this problem.

## Figures and Tables

**Table 1:** Statistical moments of signal.

Statistical Moments	Value
Mathematical expectation *m*_1_, (mA)	0.0175
Variance *m*_2_, (mA^2^)	0.0009
Skewness *A*_3_	4.0429
Kurtosis *k*_4_	17.3038

**Table 2:** Statistical moments of noise.

Realization	Mathematical Expectation m1, (mA)	Std *σ_x_*, (mA)	Shape Parameter *α*	Kurtosis *k*_4_
1	$3.9\times 10^{-5}$	$5.55\times 10^{-4}$	1.25	6.77
2	$3.6\times 10^{-5}$	$4.90\times 10^{-4}$	1.51	4.02
3	$3.4\times 10^{-5}$	$2.55\times 10^{-4}$	1.80	3.81
4	$3.1\times 10^{-5}$	$2.32\times 10^{-4}$	2.08	2.42

**Table 3:** Statistical moments for signal and noise.

Statistical Moment Order	Statistical Moments for Noise	Statistical Moments for Signal
1	$4.3\times 10^{-4}$	−5.7
2	$7.9\times 10^{-4}$	147
3	$6.9\times 10^{-5}$	$2.07\times 10^{3}$
4	$1.1\times 10^{-4}$	$5.1\times 10^{4}$
5	$2.2\times 10^{-6}$	$1.02\times 10^{6}$
6	$1.9\times 10^{-6}$	$2.2\times 10^{7}$
7	$5.6\times 10^{-8}$	$4.6\times 10^{8}$
8	$3.4\times 10^{-8}$	$9.8\times 10^{9}$
9	$1.35\times 10^{-9}$	$2.1\times 10^{11}$
10	$6.6\times 10^{-10}$	$4.4\times 10^{12}$
11	$3.15\times 10^{-11}$	$9.3\times 10^{13}$
12	$1.32\times 10^{-11}$	$1.98\times 10^{15}$

**Table 4:** Mahalanobis distance between the estimates of moments $A_{p1q1}\;and\;A_{p2q2}$ characterizing the skewness of signal and noise.

Order of Skewness	Skewness Signal	Skewness Noise	Std of Skewness Signal	Std of Skewness Noise	Mahalanobis Distance
*p* = 2, *q* = 1	−0.15	0.0013	0.043	0.016	108
*p* = 3, *q* = 2	−0.31	0.0022	0.090	0.055	43
*p* = 4, *q* = 2	−0.49	0.0035	0.143	0.16	17
*p* = 5, *q* = 2	−0.64	0.0059	0.193	0.53	15

**Table 5:** Mahalanobis distance between the estimates of moments $k_{p1q1\;}and\;k_{p2q2}$ characterizing the kurtosis of signal and noise.

Order of Kurtosis	Kurtosis Signal	Kurtosis Noise	Std of Kurtosis Signal	Std of Kurtosis Noise	Mahalanobis Distance
*p* = 1, *q* = 1/2	1.00	1.18	0.003	0.0065	3.7 × 10^3^
*p* = 3, *q* = 2	1.06	2.16	0.031	0.042	1.78 × 10^3^
*p* = 4, *q* = 2	1.12	3.00	0.051	0.11	1.37 × 10^3^
*p* = 5, *q* = 2	1.20	4.17	0.084	0.25	1.33 × 10^3^
*p* = 6, *q* = 2	1.26	5.91	0.12	0.65	1.39 × 10^3^

## Data Availability

Data available on request from the authors.

## Abbreviations

The following abbreviations are used in this manuscript:

FIR	finite impulse response
IIR	infinite impulse response
LED	light-emitting diodes
PDF	probability density function
SNR	signal-to-noise ratio
